# Hydrophilic Submicron Nanogel Particles for Specific Recombinant Proteins Extraction and Purification

**DOI:** 10.3390/polym12061413

**Published:** 2020-06-24

**Authors:** Gaëlle Levourch, Noureddine Lebaz, Abdelhamid Elaissari

**Affiliations:** 1 Unité mixte CNRS-BioMérieux, Ecole Normale Supérieure de Lyon, 46 Allée d’Italie, 69364 Lyon, France; happy.phd2020@gmail.com; 2 Univ Lyon, University Claude Bernard Lyon 1, CNRS, LAGEPP, UMR 5007, 43 Boulevard du 11 Novembre 1918, F-69100 Villeurbanne, France; noureddine.lebaz@univ-lyon1.fr

**Keywords:** nanohydrogel particles, adsorption, complexation, metal ions, recombinant protein

## Abstract

In biomedical diagnosis and bionanotechnologies, the extraction and purification of proteins and protein derivatives are of great interest. In fact, to purify recombinant proteins for instance, new methodologies and well appropriate material supports need to be established and also to be evaluated. In this work, hydrophilic nanohydrogel particles were prepared for recombinant proteins extraction for purification purpose. The prepared nanohydrogel polymer-based particles are hydrophilic below the volume phase transition temperature (TVPT) and dehydrated above the TVPT, due to the thermally sensitive poly(N-alkyl acrylamide) and poly(N-alkyl methacrylamide) derivatives. Then, the use of heavy metal ions in the presence of such functional particles should specifically capture recombinant proteins (i.e., proteins bearing a poly(histidine) part). In order to understand and to optimize the specific capture and the purification of recombinant proteins, various parameters have been investigated as a systematic study. Firstly, the adsorption was investigated as a function of pH and protein concentration. According to high hydration of the prepared nanohydrogel, no marked adsorption was observed. Secondly, the effect of pH was investigated and found to be the driven parameter affecting the metal ions immobilization and the recombinant proteins complexation. As a result, high protein complexation was observed at basic pH compared to non-complexation at acidic pH medium. The immobilized proteins via complexation were released by changing the pH. This decomplexation seems to be effective but depends on fixation conditions and particle surface structure.

## 1. Introduction

Polymer latexes have received increasing interest as supports in numerous applications, especially in the biomedical field, due to the versatility of many heterophase polymerization processes (emulsion, dispersion, microemulsion, precipitation, etc.) for making well-defined microspheres of a specific size range and surface group functionalities [1,2,3,4].

Nowadays, to enhance the sensitivity in biomedical diagnostics, the purification and the concentration steps are essential. In fact, in various biomedical domains, the limiting step is mainly related to the needed purification of a small amount of biological sample. This step has to be rapid, low cost, and easy to set down. In the case of proteins or proteic materials, this is generally performed using chromatography [5]. Unfortunately, such a method has a few drawbacks: the protein solution obtained after purification is highly diluted and the cost and the implementation are too restricting. Consequently, the challenge is to develop new tools and methodologies leading to a rapid and specific proteins purification and the alternative could be the use of colloidal latex particles [6]. The use of latex is generally due to the high specific surface (several m² per gram of particles), and their modular physicochemical properties [7].

In recent years, stimuli-responsive nanoparticles have been largely studied in the literature because of their specific properties that make them interesting candidates for a multitude of applications, especially in the biomedical field [8]. These smart materials exhibit structural and conformational changes as a consequence of their environment properties’ fluctuations (temperature, pH, ionic strength, etc.) [9,10,11]. For instance, the hydrophilic latexes are employed as solid-phase supports for the immobilization of biomolecules such as proteins or peptides in view of reducing non-specific proteins adsorption [12,13].

Specific attention has been paid to the investigation of poly-N-isopropylacrylamide (polyNIPAM) as thermally-sensitive polymer since the late 1980s [14]. This polymer exhibits a low critical solubility temperature (LCST) in the range of 30–35 °C in aqueous solution [15]. It collapses above the LCST and swells below it [16]. PolyNIPAM-based aqueous microgels were first synthesized by a surfactant-free emulsion polymerization of aqueous NIPAM and methylene-bis-acrylamide [17]. This simple method gives uniform submicron particles. To confer to the fabricated colloidal microgel particles pH additional sensitivity, microcapsules with interpenetrating polymer network structure based on polyNIPAM and poly(acrylic acid) are synthetized and characterized [18,19,20]. Moreover, core-shell gels consisting of water-insoluble core latex particles coated with a polyNIPAM shell are reported. Duracher et al. (1998) prepared and characterized monodisperse cationic polystyrene-polyNIPAM core-shell particles [21,22]. They used aminoethyl methacrylate hydrochloride (AEM) as a comonomer and methylene-bis-acrylamide as crosslinker and emphasized that the structure of the hydrophilic shell layer depends upon the polymerization process and amount of AEM used. Thermosensitive polyNIPAM coated nanomagnetic particles (Fe_3_O_4_) are also investigated and reviewed by Yi et al. (2013) [23]. Other inorganic core materials such as silica and metals are reported [24,25,26]. More recently, thermoresponsive nanoparticles with a liquid-crystalline surfactant core are prepared from the mixture of neutral block copolymer salts containing poly(ethylene oxide) and PolyNIPAM blocks as shell materials [27]. Details on the core properties that display liquid-crystalline structure may be found in [28]. Inversely, PolyNIPAM could be used as the core and coated with different organic or inorganic shells such us poly(N-isopropylmethacrylamide) [29] and silica [30].

Homogeneous and core-shell gels are tested for the sorption and desorption of proteins [31,32], enzymes [33,34], nucleic acids [35], peptides [13], and bacteria [12]. They are also used as drug carriers [36,37,38] and as antimicrobial encapsulating agents [39,40].

In this work, the idea was to evaluate the potential application of various functional thermally sensitive submicron polymer particles as a solid support for recombinant proteins purification. The chosen latexes are used as a model only. The use of colloidal particles needs the control of the possible interactions involved in the adsorption process, which may compete with the complexation. In fact, to purify proteins, only the complexation should be the driven parameter in the immobilization process. Consequently, both the adsorption and the immobilization via metal ions should be investigated as a systematic study and as a function of various physicochemical parameters such as pH, metal ion concentration, protein amount, and surface particles nature.

## 2. Materials and Methods

### 2.1. Materials

The Core 119 protein is from the capsid of the hepatitis C virus (HCV). This recombinant protein has a histidine tag on the C-terminus. Its theoretical isoelectric point is at pH = 12.01 and its molecular weight is M_w_ ≈ 14514 g.mol^−1^. This recombinant protein was purified with Ni-NTA Magnetic Agarose Beads (QIAGEN, Hilden, Germany).

Disodium hydrogen phosphate and sodium phosphate buffers were from Prolabo (Paris, France) and used to prepare phosphate buffers at different pH values. Water (deionized and deoxygenated) was of Milli-Q grade (Millipore SA, France). Sodium chloride from Prolabo and nickel (II) sulfate hexahydrate from Aldrich Chemical were used as received. Coomassie Plus Protein Assay Reagent was from Pierce and was used as a reactant to quantify protein concentration.

Styrene monomer (99% from Janssen, Beerse, Belgium) was purified by vacuum distillation and stored at −20 °C. N-isopropylacrylamide (NIPAM) (from Eastman Kodak Company, Rochester, USA) was purified via solubilization and crystallization processes using a 60/40 hexane/toluene mixture. Methylene bisacrylamide (MBA) (from Aldrich, Darmstadt, Germany) was used as a cross-linking monomer and 2-aminoethylmethacrylate hydrochloride (AEMH) (from Kodak) was used as a functional monomer. These two reactants (MBA and AEMH) were used as received. 2,2′-azobis(2-amidinopropane) dihydrochloride (V50) (from Wako, Neuss, Germany) was used as radical cationic initiator and it was recrystallized from 50/50 acetone/water mixture and dried under vacuum before use. Potassium persulfate (KPS) (reagent grade from Prolabo) was used as radical anionic initiator without further purification. N-(vinylbenzylimino)-diacetic acid (IDA) was prepared according to Morris et al.’s (1959) [41] reaction and purified before use.

### 2.2. Methods

#### 2.2.1. Preparation of Cationic and Anionic Latexes

Polystyrene core polyNIPAM shell (CS) latex was prepared using a shot-growth polymerization process. At first, batch polymerization (in 200 mL water) of styrene (18 g) and NIPAM (2 g) using V50 (0.2 g) as a cationic initiator was carried out. After 79% polymerization conversion, an aqueous solution containing NIPAM, MBA, AEMH, and V50 was injected into the preformed latex particles and the polymerization was then conducted overnight [21,22]. The detailed recipe is given in Table 1.

Nanohydrogel (HG1) was prepared via polymerization and was carried out in a 100-mL round-bottomed four necked flask equipped with a glass anchor shaped stirrer, condenser, and nitrogen inlet. Monomers (NIPAM and MBA) dissolved in boiled and deoxygenated water were then added. After temperature equilibrium (80 °C), the solution was stirred for 30 min at polymerization temperature before introducing the initiator (KPS) dissolved in water. The solution was stirred at a constant rate under nitrogen during polymerization and the reaction was carried out during 6 h. The recipe is given in Table 1.

Regarding the second nanohydrogel (HG2), it was prepared following the same process as for HG1 via polymerization and was carried out in a 100-mL round-bottomed four necked flask equipped with a glass anchor shaped stirrer, condenser, and nitrogen inlet. Monomers (NIPAM, IDA, and MBA) dissolved in boiled and deoxygenated water were then added. After temperature equilibrium (80 °C), the solution was stirred for 30 min at polymerization temperature before introducing the initiator (KPS) dissolved in water. The solution was stirred at a constant rate under nitrogen during polymerization and the reaction was carried out at 80 °C during 6 h. The recipe is given in Table 1 as well.

#### 2.2.2. Characterization of Latexes

The prepared polymer particles were cleaned by repetitive centrifugation and redispersion in deionized water in order to remove the free water-soluble polymer and the free electrolytes before any characterization.

Particle size distributions were determined by quasi-elastic light scattering (QELS, N4 from Coultronics, France). Electrophoretic mobility of highly diluted latex particles in 10^−3^ M NaCl solution was measured as a function of pH and temperature from 20 to 50 °C using Zetasizer 3000HS (Malvern Instruments, Grovewood, UK) in order to examine the influence of both pH and temperature on surface charge density variation.

The shape of the particles was observed by scanning electron microscopy (SEM, Hitachi S 800 (Ueden, Germany), CMEABG at Claude Bernard University, Lyon I, France). Samples for SEM were prepared by placing a drop of the dispersion directly onto an aluminum sample holder and the latex drop was dried at room temperature. All specimens for SEM measurements were sputtered with gold at fixed conditions (time 150 s, current 20 mA, voltage 2 kV). A standard voltage (10 kV) was used for SEM experiments.

#### 2.2.3. Adsorption and Complexation of Protein onto Latex Particles

Adsorption and complexation experiments were performed in 10-mM phosphate buffers and at a given pH (ranging between 5 to 9). The data reported in this study are the average values of duplicate or triplicate experiments. The adsorption study was performed without adding nickel ions.

The amount of protein adsorbed was determined via depletion method by quantifying residual free proteins in the supernatant after removing polymer particles using a centrifugation step (13,000 rpm for 30 min at 20 °C). The concentration of protein molecules in the supernatant was determined using the Bradford’s method [42] based on the calibration curve.

#### 2.2.4. Complexation as a Function of pH

For the complexation experiments, latex particles and 10^−2^ mM of nickel ions were first incubated at 20 °C during 15 min in phosphate buffer solutions at various pH (5 to 9) to allow the complexation of nickel ions on colloidal particles first. Thereafter, proteins and particles-nickel ions were mixed and incubated at 20 °C for 2 h. After the incubation time, the amounts of immobilized protein were determined as described above.

#### 2.2.5. Complexation as a Function of Nickel and Protein Concentrations

In order to study the influence of nickel ions concentration on the protein complexation at various pH and at 20 °C, different quantities of nickel (ranging from 10^−4^ to 10^−1^ mM) were added to the colloidal particles containing systems. The same procedure described above was followed for investigating the influence of protein concentration after adding various quantities of protein ranging between 7 and 35 mg per gram of polymer particles.

#### 2.2.6. Decomplexation Study

The release from the complexation was studied as a function of pH (fixation at neutral or basic pH and removal at acidic pH). The experiments were performed as follows: first, protein adsorption was performed at neutral or basic pH (ranging from 7 to 9) at 20 °C. Secondly, the protein-nickel-latex particle complexes were separated from the medium and redispersed in the same volume of acidic phosphate buffer solution (pH = 5 or 6). Release from complexation was then carried out at 20 °C under magnetic stirring (at 900 rpm). Finally, the amount of released protein was determined by measuring the free protein concentration in the supernatant as described above.

Due to the complex nature of the used latexes, the amounts of protein adsorbed or complexed were expressed in mg of adsorbed protein per g of latex particles rather than in mg per m².

## 3. Results and Discussion

### 3.1. Characterization of the Particles

#### 3.1.1. Scanning Electron Microscopy Analysis

**Figure 1:**
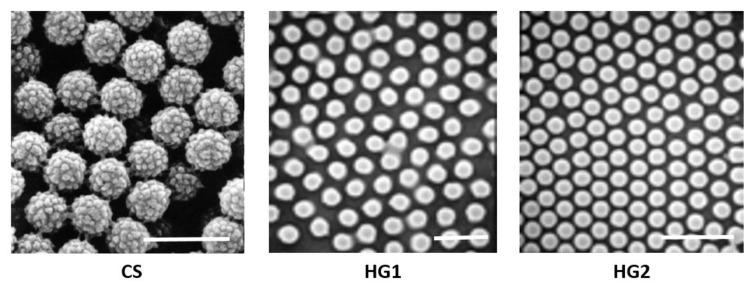
Scanning Electron Microscopy (SEM) analysis of the prepared particles (scale bar is 1 μm).

Scanning Electron Microscopy (SEM) was first performed and the obtained images show that all particles are spherical and seem also to be submicronic and narrowly distributed in size (Figure 1). The real size cannot be determined from SEM images for such particles. In fact, the platteness of such soft particles induces an overestimation of the particle size as reported by Hazot et al. (2003) [43].

#### 3.1.2. Hydrodynamic Particle Size

**Figure 2:**
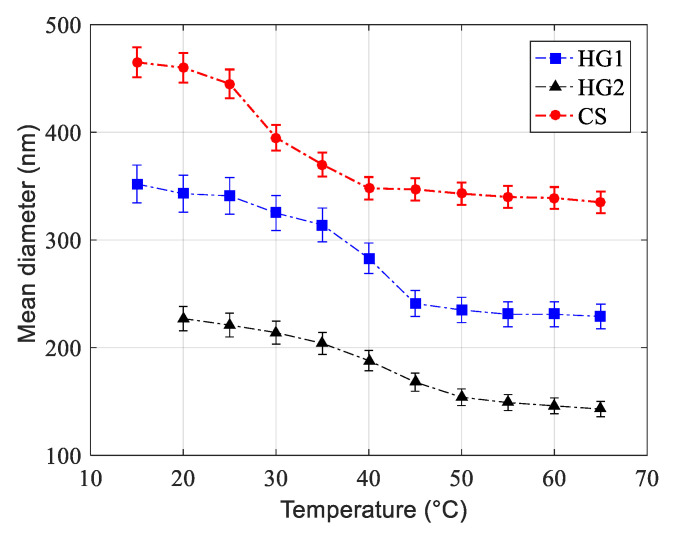
Hydrodynamic mean particle size of HG1, HG2, and core shell (CS) as a function of temperature in 1 mM NaCl solution.

Hydrodynamic mean particle size variation as a function of temperature measured by QELS in 10^−3^ M NaCl solution is reported in Figure 2 and Table 2 for all dispersions. The measured hydrodynamic mean particle size (z-average) for all latexes decreases with increasing the temperature from 15 to 70 °C, reflecting changes in the shrinkage of these soft particles. For the core-shell particles (CS), the volume phase transition was found to be close to the corresponding LCST (≈32 °C) of pure polyNIPAM in a salt free medium. Regarding HG1 and HG2, the drastic changes were found to be close to 40 °C for both of them (transition temperature of the non-crosslinked polyNIPMAM homopolymer: 44 °C). Similar results have already been reported by Kawaguchi and al. (1992) [44] for cross-linked polyNIPAM nanohydrogel particles and by Duracher and al. (1998) [21] for polyNIPMAM nanohydrogel particles.

#### 3.1.3. Electrophoretic Mobility

**Figure 3:**
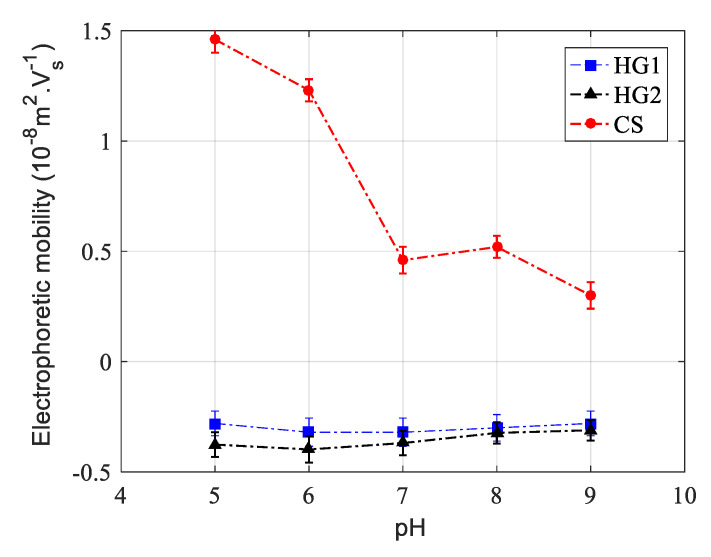
Electrophoretic mobility of CS, HG1, and HG2 as a function of pH at 25 °C and in 10 mM phosphate buffer.

The electrophoretic mobility of all dispersions was measured as a function of pH in a 10-mM phosphate buffer and at 25 °C, and the obtained results are reported in Figure 3. For the core-shell-like particles, the measured electrophoretic mobility exhibits a positive character in the studied pH domain ranging from pH = 5 to pH = 9. The positive electrophoretic mobility can be attributed to the cationic character of both V50 used as initiator and AEMH used as functional monomer. Electrophoretic mobility decreases as a function of pH due principally to deprotonation of both V50 and AEMH. A similar tendency has been reported by Duracher et al. (1998) [21].

For HG1 and HG2 nanohydrogels, a constant and negative electrophoretic mobility is observed in the investigated pH range, reflecting the negative character and low surface charge density attributed mainly to the presence of sulfate groups originating from KPS used as the initiator in the polymerization recipe. The slight difference between HG1 and HG2 can be attributed to the presence of both carboxylic and sulfate groups on HG2 nanogel particles. However, the difference is not significant regarding experimental uncertainty as reported in Figure 3.

**Figure 4:**
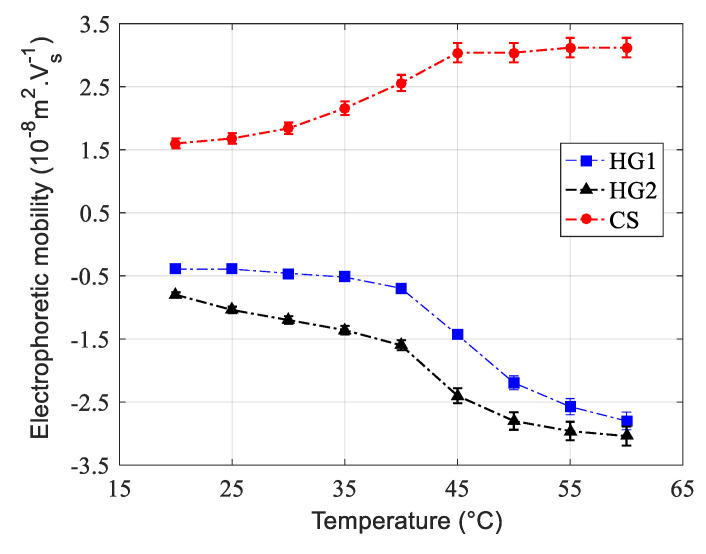
Electrophoretic mobility of HG1, HG2, and CS as a function of temperature at pH = 7 in a 10-mM phosphate buffer.

Electrophoretic mobility was also investigated as a function of temperature. The obtained results are reported in Figure 4 for all dispersions. For the three dispersions, the measured electrophoretic mobility increases (in absolute value) with increasing temperature. This is attributed to changes in the hydrodynamic size as a function of temperature. In fact, with increasing temperature, the hydrodynamic size decreases, leading to an increase in the surface charge density and consequently to an increase in the electrophoretic mobility. Similar tendency has been already reported by Pelton and Chibante (1986) [17], Kawaguchi et al. (1992) [44], and then Nabzar et al. (1998) [45] for polyNIPAM microgel particles.

### 3.2. Protein Immobilization

#### 3.2.1. Protein Adsorption

**Figure 5:**
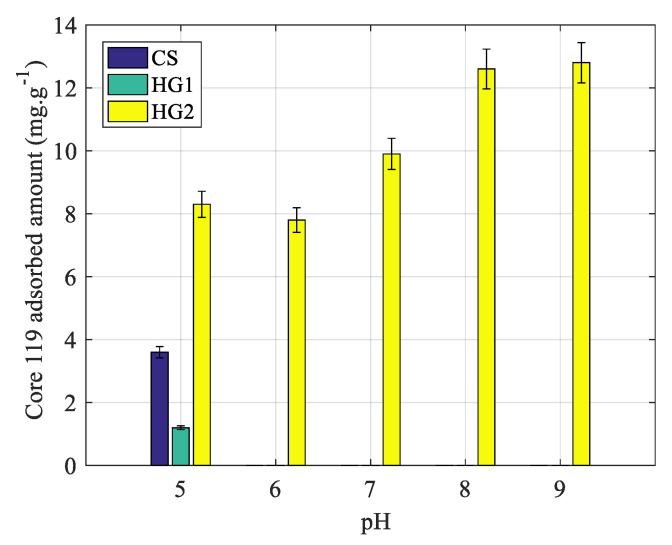
pH influence of Core 119 adsorption onto CS, HG1, and HG2 latexes. Conditions: phosphate buffer at different pH (from 5 to 9), ionic strength 10 mM, adsorption temperature 20 °C.

Protein adsorption onto all particles has been investigated as a function of pH, in a 10-mM phosphate buffer and at 20 °C. The obtained results are reported in Figure 5. As well known, proteins adsorption onto thermally sensitive N-alkylacrylamide derivatives based particles, and on hydrated surfaces is low and even undetectable [46]. Whereas, the adsorption on dehydrated charged nanohydrogels above the volume phase transition temperature is attributed to the dehydration process which induces both hydrophobic and electrostatic interactions [47]. In the case of the present study, the adsorption results show that adsorption is high for HG2 compared to CS and HG1 irrespective of pH. This can be attributed to the possible presence of hydrogen binding between protein and nanogel particles. For CS and HG1, the non-observed adsorption above pH = 5 can be attributed to the total absence of both hydrophobic and attractive electrostatic interactions. Whereas, the slight adsorbed amount at pH = 5 can be attributed to the possible hydrogen binding adsorption process.

In brief, the low protein adsorbed amounts on CS and HG1 are in the sensitivity limit of the measurement method since the quantities are lower than 5 mg/g. Consequently, the adsorbed amount at 20 °C can be almost considered as negligible for both nanogels (CS & HG1) irrespective of pH in the investigated range.

#### 3.2.2. Protein Complexation

As well-known, Immobilized Metal Affinity Chromatography (IMAC)-like approaches using Nickel-Nitriloacetic Acid (Nickel-NTA) and nickel ions as the active site for specific histidine immobilization has been largely reported. The Ni^2+^ ion has six coordination links with four requested for nickel ion immobilization on the support (i.e., NTA compound) and two remain links for nitrogen atoms of the side chain cycle of two histidine residues immobilization. This motivated the use of Ni for protein complexation in this study.

##### Effect of pH

**Figure 6:**
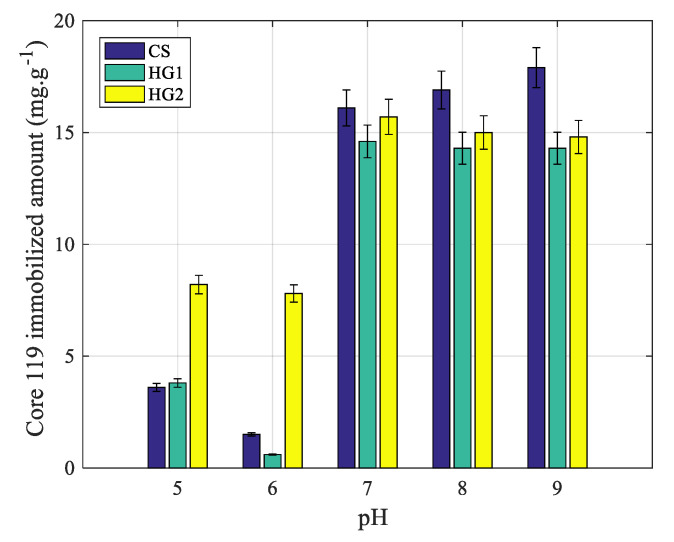
pH dependence of Core 119 fixation onto CS, HG1, and HG2 in presence of nickel. Conditions: phosphate buffers at different pH (from 5 to 9), ionic strength 10 mM, adsorption temperature 20 °C, [Ni] = 10^−2^ M.

As already reported, the protein adsorption below the volume phase transition temperature of poly(N-alkylacrylamide) derivative nanohydrogel revealed low and in some cases no adsorption irrespective of pH and salinity. Then, the complexation study was investigated as a function of pH, in 10-mM phosphate buffer and at 20 °C (i.e., below the volume phase transition temperature of all prepared nanohydrogels). The obtained results are reported in Figure 6. The presence of Ni ions enhanced the protein immobilization principally at basic pH for CS and HG1. In fact, at basic pH, the adsorption was found to be negative, whereas, the complexation was found to be highly marked. Surprisingly, for HG2, it is not possible to discriminate between adsorption and complexation since the same fixation amounts are observed during adsorption as discussed above. This unexpected result can be attributed to the chemical structure of the nanohydrogel. It is interesting to notice that the protein immobilized amounts on CS and HG1 at acidic pH are low and in the detection limit of proteins in the supernatant. Consequently, the protein complexation via imidazole groups in the presence of Ni ions can be considered effective on CS and HG1 compared to HG2 and special attention will be more dedicated to CS and HG1.

##### Effect of Nickel Concentration on Proteins Immobilization

**Figure 7:**
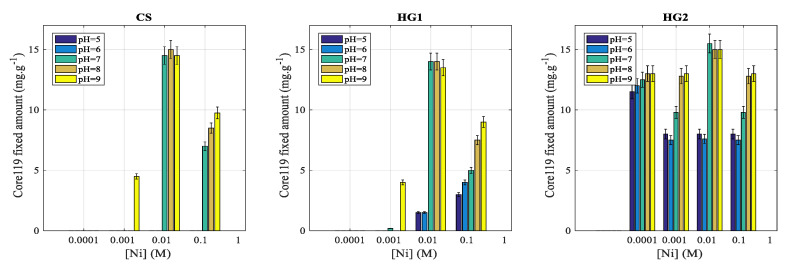
Effect of nickel concentration onto Core 119 fixation for core-shell (CS) particles, nanohydrogels HG1 and HG2. Conditions: phosphate buffers at different pH (ranging from 5 to 9), at 20 °C and for various [Ni] from 10^−4^ M to 10^−1^ M.

The goal of this part is to investigate the influence of nickel concentration and pH on the complexation of a recombinant protein. Nickel concentration was then varied from 10^−4^ M to 10^−1^ M following the same methodology as described above. The obtained results are reported in Figure 7.

For HG2, the immobilized amount is found to be constant irrespective of Ni concentration and pH. The observed complexation on CS and HG1 can be attributed to nickel atoms coordination on oxygen and nitrogen atoms present on the interfacial polymer structure. Surprisingly, for both core-shell (CS) and nanohydrogel (HG1), the observed complexation amounts are almost the same above pH = 7. Whereas, below pH = 7, the immobilization was found to be more marked on HG1 compared to CS. The maximum complexation efficiency was found to be for 10-mM nickel ions concentration. It is interesting to notice that the colloidal stability was maintained irrespective of nickel ions concentration. This is due to steric stabilization of the prepared nanohydrogel dispersions.

#### 3.2.3. Effect of Protein Concentration

**Figure 8:**
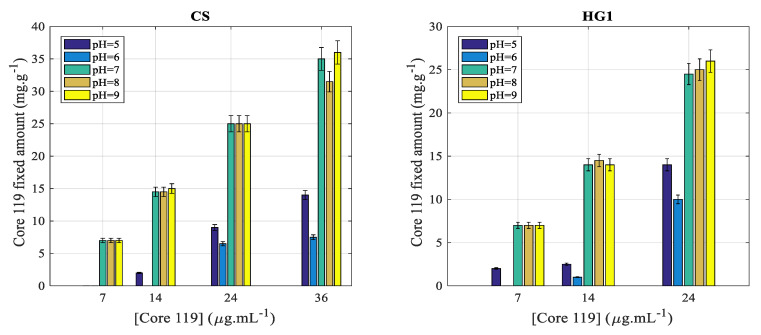
Effect of protein concentration on the complexation amount for CS and HG1 at pH ranging from 5 to 9. The complexation was performed at 20 °C and in the presence of 10-mM nickel ions concentration 3.3. Protein extraction study.

The effect of protein concentration on the complexation of HG2 was discarded since the adsorption was more pronounced irrespective of pH and nickel ions concentration. Then, the aim of this part is the investigation of the effect of protein concentration on the complexation efficiency on core-shell (CS) particles and on HG1 nanohydrogel particles. The obtained results are reported in Figure 8 with a 10^−2^ M Nickel concentration.

As it can be seen in Figure 8, the protein complexation amount increases with increasing the initial protein concentration. Similar tendency and almost the same complexation amounts are observed for both CS and HG1. For pH ≤ 6, the complexation efficiency is almost close to zero and even negligible, but increases slightly with increasing protein concentration. Whereas, above pH = 6, the complexation amount was found to be high and increases as a function of initial protein concentration.

Interestingly, for pH ≥ 7, the complexation amount increases almost linearly with increasing the initial protein concentration in the medium.

In this part, the release of complexed proteins on HG1 and CS particles was investigated by performing the complexation at pH ≥ 7 and the release at acidic pH at which the complexation was found to be less effective. In addition, the effect of imidazole on protein release was also investigated.

In order to estimate the real extracted amount, the adsorbed amount was subtracted, leading consequently to the purification yield (% efficiency was deduced from proteins amount released with respect to initial proteins amount).

**Figure 9:**
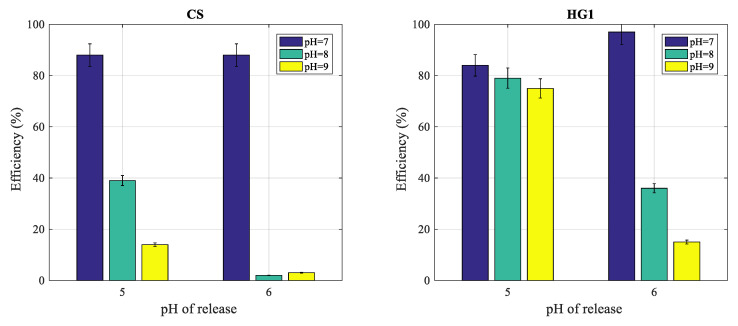
Effect of pH of fixing and that of salting out on protein release from CS (left) and HG1 (right) latexes. Conditions: phosphate buffers at different pH (5 and 6), temperature 20 °C, [Ni] = 10^−2^ M.

The protein complexation on (1 mg/mL) polymer particles was first performed at a given pH (7, 8 and 9) in the presence of 10^−2^ M nickel ions concentration (optimal concentration as above described), at 20 °C and the release was performed after removing the supernatant and replaced by equivalent volume of 10-mM phosphate buffer (pH = 5 or pH = 6). The obtained results are reported in Figure 9 for both polymer particles.

For CS (Figure 9, left), protein extraction after complexation at pH = 7 leads to 85% extraction efficiency at pH = 5 and pH = 6. Whereas, the complexation performed at pH = 8 or pH = 9 leads to low protein extraction yields at both releases pH (5 and 6).

For HG1 (Figure 9, right), the extraction yields are found to be around 80% when the release was performed at pH = 5 and irrespective of complexation pH. As for CS, the extraction at pH = 6 after complexation at pH = 5 leads to high extraction yield. Whereas, the release after complexation at pH = 8 and 9 leads to low extraction yield (below 40%).

## 4. Conclusions

Nanohydrogel polymer particles were prepared via radical polymerization using charged initiator. The particles are spherical in shape and narrowly size distributed with a hydrodynamic mean diameter between 100 nm and 500 nm. The effect of temperature on hydrodynamic particle size was investigated, revealing the swelling and deswelling of the particles, and consequently, the hydrogel character of the prepared polymer particles.

The adsorption of a recombinant protein on the three nanogel particles was investigated at 20 °C (i.e., below the volume phase transition of each particle). The adsorption was found to be negligible for negatively charged polystyrene core/crosslinked polyNIPAM shell and also on classical polyN-isopropylmethacrylamide nanogel (<5 mg/g). Whereas, the adsorption of carboxylic containing polyN-isopropylmethacrylamide nanogel was not found to be more marked due to the possible hydrogen interactions. The complexation of recombinant proteins via nickel ions was performed on CS and HG1 and found to be pH dependent. Whereas on HG2, it was not possible to discriminate between adsorption and complexation. The feasibility of recombinant protein extraction was performed in CS and HG1 and high extraction yields (≥80%) were obtained principally when the complexation was performed at basic pH and the release at pH = 5 or pH = 6.

As a perspective of this work, it will be possible to scale-up the process for nanogel preparation and complexation. The possible limiting use of protein extraction will be related to the centrifugation step for phases separation in the case of such polymer-based nanohydrogels. But if the physical chemistry properties of the nanohydrogels are transferred on magnetic bead, then complexation, the extraction, and the purification processes will be easy to perform by using a single permanent magnet.

## Figures and Tables

**Table 1:** Recipe of core-shell and nanohydrogel particles preparation.

Product (g)	CS	HG1	HG2
Water (mL)	200	50	50
NIPAM	5.07 ^*^	1	1
AEMH	0.147 ^**^	/	/
IDA	/	/	0.012
MBA	0.069 ^**^	0.120	0.120
V50	0.122 ^**^	/	/
KPS	/	0.012	0.012

^*^ Batch part was performed using 18 g of styrene, 2 g NIPAM and 0.2 g V50. ^**^ For shot grow process.

**Table 2:** Colloidal characteristics of cationic core-shell and anionic nanohydrogel latexes. Hydrodynamic mean particle size (z-average) was determined by quasi-elastic light scattering (QELS); the thermally-sensitive nanohydrogel hydrated thickness layer is calculated as (δ = (D_20°C_ − D_50°C_)/2).

Sample	D_h_ (20 °C) (nm)	D_h_ (50 °C) (nm)	δ (nm)
CS	460	343	58.5
HG1	350	225	62.5
HG2	220	140	40.0
